# Travelers' health.

**DOI:** 10.3201/eid0403.980315

**Published:** 1998

**Authors:** M Cetron, J Keystone, D Shlim, R Steffen

**Affiliations:** Centers for Disease Control and Prevention, Atlanta, Georgia, USA.


### Travelers' Health

**Martin Cetron,* Jay Keystone,† David Shlim,‡ and Robert Steffen§**
*Centers for Disease Control and Prevention, Atlanta, Georgia, USA; †The Toronto Hospital, Toronto, Ontario, Canada; ‡The Canadian International Water and Energy Consultants Clinic Travel Medicine Center, Kathmandu, Nepal; §University of Zurich and World Health Organization Collaborating Centre for Travelers' Health, Zurich, Switzerland

Over the last century and a half, the world's population has grown from one billion to almost six billion, and the time required to circumnavigate the globe has decreased from 365 days to fewer than 3. Currently, 1.4 million persons travel internationally by air every day. The speed and volume of international travel are cited by the Institute of Medicine as principal factors contributing to the global emergence of infectious diseases. This panel assessed the effect of travel on emerging infections from the perspectives of both industrialized (Jay Keystone) and developing countries (David Shlim), discussed the role of vaccination in preventing travel-related infections (Robert Steffen), and explored unique health issues associated with travel into space (David Shlim and Thomas Marshburn, National Aeronautics and Space Administration [NASA]).

### Travel and the Spread of Emerging Infectious Diseases

In the past decade, international travel has grown dramatically to the point where more than 500 million travelers cross international borders annually by commercial aircraft alone. Mass migrations of refugees, workers, and displaced persons have led to a steady growth of urban centers at the expense of rural areas. These population movements have been ideal conduits for the global spread of new and reemerging infectious diseases. Virtually any place in the world can be reached within 36 hours, less than the incubation period for most infectious diseases. Infectious agents can spread from person to person directly or to vectors at the traveler's destination. Vehicles of human transport, such as aircraft and ships, also transport the infectious agent and its vector. In addition, mass migrations have facilitated the rapid and immediate spread of communicable disease among refugees and displaced persons.

If travelers are responsible for the spread of infectious agents, they are also ideal sentinels for the arrival of an infectious agent in a new community. In 1969, the first documented outbreak of Lassa fever was noted among American missionaries in Lagos, Nigeria. In 1992, two Peace Corps volunteers contracted neuroschistosomiasis; a subsequent investigation confirmed that Lake Malawi was an important source for the transmission of *Schistosoma haematobium.* More recently, chloroquine-resistant *Plasmodium vivax* was documented for the first time in North Africa in U.S. Army troops returning from Somalia.

These and many other examples show that travel is instrumental in the spread of new and reemerging infectious diseases. As international travel grows by more than 10% per year and major population shifts continue because of political, economic, and social instability, public health agencies will have to focus their infectious disease surveillance programs on travelers, migrants, and the vehicles of transport. Recognition that travelers are important sentinels for emerging infectious diseases has stimulated the International Society of Travel Medicine and the Centers for Disease Control and Prevention to carry out a joint surveillance project, Geosentinel, in 22 travel clinics around the world.

### Vaccinating Travelers

Vaccination is only one of several strategies of prophylaxis in travel medicine. Selection of immunizations should be based on requirements and on risk for infection. According to the International Health Regulations, many countries require proof of yellow fever vaccination on the International Certificate of Vaccination. The revised International Health Regulations, to be submitted to the World Health Assembly next year, will likely maintain this requirement. Additionally, a few countries still require proof of vaccination against cholera, diphtheria, and meningococcal disease under specific circumstances.

Recommended immunizations often are more important for travelers' health than the required or routine ones. The most frequent vaccine-preventable infection in travelers to developing countries is hepatitis A, with an average incidence rate of 0.3% per month in susceptible persons; in high-risk backpackers or foreign-aid volunteers, this rate is 2.0%. The World Health Organization and many national expert groups recommend that all travelers visiting developing countries be immunized against hepatitis A. Several other immunizations are recommended for special risk groups. Hepatitis B is a problem among expatriates, particularly if they live close to the native population or engage in high-risk behavior (e.g., unprotected casual sex or intravenous drug use); rabies is also a problem, with approximately 0.2% to 0.4% of long-term residents bitten by animals each month—travelers to selected geographic regions with high endemicity (Southeast Asia) and exposure risk profiles warrant rabies vaccination; typhoid fever (often diagnosed among travelers to the Indian subcontinent, North and West Africa—except Tunisia—and Peru) poses a higher risk in long-term residents and in those who consume food and beverages prepared under substandard hygienic conditions (estimated incidence 0.03% to 0.003% per month); anecdotally, meningococcal disease, Japanese encephalitis, and tick-borne encephalitis have been reported in travelers (monthly incidence less than one per million travelers); immunizations against cholera and tuberculosis are only rarely recommended.

In the not-too-distant future, travelers will be offered a variety of oral vaccines against pathogens causing travelers' diarrhea, including all enterotoxigenic *Escherichia coli* as well as *Campylobacter* and *Shigella*. Additionally, immunization against Lyme disease and dengue fever will be available within the next years; vaccines against malaria and AIDS, the infections causing most death in travelers, are much further in the future.

### Emerging Diseases in a Developing Country: Observations from a Travel Medicine Clinic

The CIWEC Clinic Travel Medicine Center was the world's first self-supporting destination travel medicine clinic. Started in 1982 to provide western medical care for expatriates and tourists in Nepal, the clinic has set a standard for investigating disease risks for tourists in a developing country.

Nepal (population 22 million, resident foreign population 3,000) has an annual influx of approximately 250,000 non-Indian tourists a year. The Travel Medicine Center is situated in the capital, Kathmandu, through which 90% of tourists enter and exit the country; a high percentage of expatriates and tourists use the clinic for medical care, creating a favorable situation for health-risk studies.

Emerging diseases can be divided into three categories: 1) truly new diseases, such as diarrhea caused by *Cyclospora*; 2) newly noted or increasing diseases, such as meningococcal meningitis and Japanese encephalitis; and 3) unexplained illnesses, such as a cluster of cases of sudden death among young trekkers in teahouses. The Travel Medicine Center has made it possible to observe all three categories. Most of the above conditions would likely have remained unnoticed and unreported were it not for the presence of a clinic focused on the health risks of foreigners in Nepal.

The value of a research-oriented travel medicine center in Kathmandu extends beyond the borders of Nepal. Research into diarrheal disease, hepatitis, typhoid fever, rabies immunoprophylaxis, and altitude illness has been relevant to other locations. Specific disease risks for travelers to Nepal have been carefully defined. Clinics in other developing countries should be identified, and similar data gathering and observations should be encouraged.

### Travel Medicine in the 21st Century: NASA Perspectives

Space travel entails unique circumstances that influence the health of astronauts, most notably, the limitations imposed by zero-gravity environments. The impact of weightlessness on human physiology is substantial and affects various body systems: neurologic, psychologic, vestibular, cardiovascular, musculoskeletal, endocrine, hematologic, and immunologic. Prominent sequelae of prolonged weightlessness may include profound motor weakness and 1% to 2% loss of mineralized bone for each month in orbit; these effects can be countered by maintaining a vigorous daily exercise program while in orbit. In addition, recent studies of the immune system have documented a decrease in cell-mediated immunity among astronauts spending extended time in space.

Environmental stressors (prolonged exposure to increased temperatures, high-velocity travel [17,000 miles per hour], and prolonged confinement to limited physical space) may also affect the health of space travelers. The space environment also challenges the growth of microorganisms. Increased antimicrobial resistance has been noted among *E. coli* and *Staphylococcus aureus* bacteria in space, and fungal overgrowth can be a problem because of changes in humidity and pockets of increased condensation aboard spacecraft and space stations.

The primary strategies used by NASA to prevent health-related mishaps associated with space travel include selecting exceptionally fit, healthy crew members and imposing preflight protective quarantine. Access to the astronauts is restricted to family members beginning 10 days before departure, and a strict 7-day prelaunch quarantine is imposed.

Thus far, infectious diseases have rarely been an important problem at NASA; launch delays due to infectious diseases have occurred in only two Apollo flights and one shuttle mission. One instance of infectious prostatitis in an astronaut on the MIR space station resulted in the premature termination of his stay. Recent forays into long-term habitation in space such as those aboard MIR (3 to 6 months) will provide new insights into the feasibility of extended space travel and human colonization beyond our planet.

